# COVID-19-Induced Disruptions of School Feeding Services Exacerbate Food Insecurity in Nigeria

**DOI:** 10.1093/jn/nxab100

**Published:** 2021-05-24

**Authors:** Kibrom A Abay, Mulubrhan Amare, Luca Tiberti, Kwaw S Andam

**Affiliations:** International Food Policy Research Institute (IFPRI), Washington, DC, USA; International Food Policy Research Institute (IFPRI), Washington, DC, USA; University of Laval and Partnership for Economic Policy, Canada; International Food Policy Research Institute (IFPRI), Washington, DC, USA

**Keywords:** COVID-19, school feeding services, food security, panel data, Nigeria

## Abstract

**Background:**

The coronavirus disease 2019 (COVID-19) pandemic and associated lockdown measures have disrupted educational and nutrition services globally. Understanding the overall and differential impacts of disruption of nutritional (school feeding) services is critical for designing effective post-COVID-19 recovery policies.

**Objectives:**

The aim of this study was to examine the impact of COVID-19-induced disruption of school feeding services on household food security in Nigeria.

**Methods:**

We combined household-level, pre-COVID-19 in-person survey data with postpandemic phone survey data, along with local government area (LGA)–level information on access to school feeding services. We used a difference-in-difference approach and examined temporal trends in the food security of households with and without access to school feeding services. Of the sampled households, 83% live in LGAs with school feeding services.

**Results:**

Households experienced an increase in food insecurity in the post-COVID-19 survey round. The share of households skipping a meal increased by 47 percentage points (95% CI: 44–50 percentage points). COVID-19-induced disruptions of school feeding services increased households' experiences of food insecurity, increasing the probability of skipping a meal by 9 percentage points (95% CI: 3–17 percentage points) and the likelihood of going without eating for a whole day by 3 percentage points (95% CI: 2–11 percentage points). Disruption of school feeding services is associated with a 0.2 SD (95% CI: 0.04–0.41 SD) increase in the food insecurity index. Households residing in states experiencing strict lockdown measures reported further deterioration in food insecurity. Single mothers and poorer households experienced relatively larger deteriorations in food security due to disruption of school feeding services.

**Conclusions:**

Our findings show that COVID-19-induced disruptions in educational and nutritional services have exacerbated households’ food insecurity in Nigeria. These findings can inform the designs of immediate and medium-term policy responses, including the designs of social protection policies and alternative programs to substitute nutritional services affected by the pandemic.

## Introduction

The coronavirus disease 2019 (COVID-19) pandemic and associated lockdown policies have disrupted educational, health, and nutrition services globally, with enormous implications for households’ and children's well-being (1–4). As the spread of the pandemic increased, more than 190 countries implemented countrywide school closures, affecting 1.6 billion children globally (5). National school closures may endanger child learning outcomes, as well as children's and households’ welfare (1, 6–8). Recent simulation studies show that school closure could result in a loss of 0.3 to 0.9 y of schooling (1). [Recent studies found that school closures in Sierra Leone as a short-term policy response to the Ebola epidemics led to a drop in girls’ school enrollment by 17 percentage points (9). These studies also show that these impacts are long lasting, and hence visible postpandemic.] In addition to the direct effects on learning, school closures are likely to affect households’ food security by disrupting school feeding services that were directly contributing to households’ food security. In many countries, school feeding services represent the cornerstone of education programs and nutrition policies, and several studies have shown that school feeding programs improve the educational outcomes, gender equality, nutrition, and food security of children and their families (10–19). Understanding both the overall and differential impacts of the COVID-19 pandemic and the associated disruptions of school feeding services is critical for designing effective post-COVID-19 recovery policies and options.

This paper aims to quantify the impacts of COVID-19-induced disruptions of school feeding services on households’ food security in Nigeria. In response to the spread of the pandemic, Nigeria implemented nationwide school closures across all 37 states [including the Federal Capital Territory (FCT) of Abuja] mid-March 2020. As national school closures and disruption of school feeding programs were introduced abruptly, the situation represents a suitable natural experiment to test the causal effect of the pandemic and the associated disruptions of school feeding services on households’ food security.

We also aim to shed light on the differential impacts of school closure and disruption of school feeding services on various groups of households. For example, poorer households are more likely to rely on school feeding services for accessing nutritious diets and are likely to be disproportionally affected by the closure of school feeding programs. National school and daycare closures have significantly increased childcare and learning support needs, and working mothers or single mothers are more likely to bear the brunt of this (20). Because of these trends, evolving studies argue that the pandemic may increase existing gender inequalities (20–22).

Despite some anecdotal evidence and speculative hypotheses on the sector- and household-level impacts of COVID-19 and associated government responses, rigorous empirical studies based on household-level survey data have yet to be published. Thus, this paper contributes fresh empirical evidence on the effects of (COVID-19-induced) disruption of school feeding programs on households’ food security.

## Methods

### Country context

As part of the efforts to reduce poverty and child malnutrition, the Federal Government of Nigeria developed a social reform agenda (23–25) that includes the National Social Investment Program (NSIP). The National Home-Grown School Feeding Program (NHGSFP) is 1 NSIP intervention under this social reform agenda. The government developed a road map for implementing the NHGSFP across Nigeria in May 2014, and the program was officially launched in June 2016 (26).

The government's motivation for establishing the NHGSFP was to deliver a government-led, cost-effective school feeding program, using smallholder farmers and local procurement to enhance growth in the local economy. Although the main focus of this food-based safety net program is providing nutrition and quality food to children, the program is also designed to indirectly improve the food security of beneficiary households (26, 27). The envisioned welfare outcomes of the NHGSFP are expected to accrue to numerous stakeholders. For instance, schoolchildren will enjoy a hot, fresh, nutritionally balanced school meal; farmers or farming households (or farming cooperatives) will take advantage of enhanced access to school feeding markets; and new job opportunities will be available to communities across several supply chains, including catering, processing, and food handling (26). [Besides the direct benefits, the NHGSFP is expected to act as a vital facilitator to motivate *1*) agriculture-nutrition policies, given the direct nutritional components of NHGSFP menus; and *2*) smallholder market participation, with spillover effects on broader public agriculture commodity procurement.] The program provides 1 meal per day for each primary school child (grades 1–3) enrolled in government-owned primary schools in the implementing state and local government area (LGA). Currently, over 9 million pupils benefit from this program (19). However, the NGHSFP does not cover all LGAs in Nigeria, and some LGAs do not yet have access to school feeding services, a variation we exploit in this paper.

Nigeria was among the first African countries to record a COVID-19 case in late February 2020. As part of the measures to contain the spread of the pandemic, the federal- and state-level governments introduced various measures, including travel bans, closure of schools and religious institutions, bans on public and social gatherings, and curfew hours restricting the movement of people. In particular, the federal government announced the closure of schools and other lockdown and social distancing measures in mid-March 2020 (28). School closures were immediately implemented across all 37 states (including the FCT of Abuja) in Nigeria, such that that over 9 million pupils who were benefiting from the NGHSFP were no longer getting school meals because of the pandemic.

### Data and data sources

Our main data came from the Living Standards Measurement Study-Integrated Agriculture Survey (LSMS-ISA) for Nigeria, collected by the Nigerian Bureau of Statistics and the World Bank. We use 2 rounds of longitudinal household surveys: 1 pre-COVID-19 in-person survey and 1 post-COVID-19 phone survey. The pre-COVID-19 data are nationally representative and provide detailed information on households’ characteristics, food security, and employment outcomes. Most of the information from the pre-COVID-19 survey was collected in January and February 2019, while the post-COVID-19 phone survey data were collected between April and May 2020. The post-COVID-19 phone survey aimed at tracking households interviewed during the 2019 LSMS-ISA survey. Out of the total sample of households (4976) interviewed in the latest (2019) round, 99.2% of them provided phone numbers. Out of those households with phone numbers, a random sample of 3000 households was selected for the phone survey. The phone survey managed to successfully contact 69% of sampled households, and 94% (1950) of these households were successfully interviewed (29, 30). These 1950 households represent our final sample, and information from the phone survey was merged with information from the previous round to create a household-level panel data set. We then kept those households with complete information in both rounds.

We compiled data on COVID-19 cases and lockdown measures from the Nigerian Centre for Disease Control (30). To exploit spatial variations in access to school feeding programs, we compiled LGA-level (subdistrict) information on access to school feeding services. These data come from the Federal Ministry of Humanitarian Affairs, Disaster Management and Social Development. By January 2020 (immediately before the COVID-19 pandemic), 714 of the 774 LGAs were implementing the NHGSFP. Each state designs and implements the program in a manner suited to its own context, channeling resources through LGAs to participating schools. This disaggregated (LGA-level) variation in access to school feeding services provides an interesting source of variation that can be used to evaluate the impact of disruption of school feeding services.

Attrition in the post-COVID-19 phone survey is likely to be systematic, necessitating the need to construct and apply appropriate sampling weights to make inferences and compute nationally representative statistics. Construction of these weights should also consider sampling weights from the pre-COVID-19 survey. Considering these and going through several steps, the LSMS-ISA team constructed the sampling weights for the phone survey data to account for potential systematic attrition. Detailed discussions about these steps were provided previously (29,30).

**FIGURE 1:**
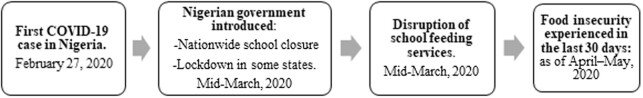
The linkage between COVID-19, disruptions in school feeding services, and food insecurity.

**Figure 1** conceptualizes the linkages and relationships we aim to test in this paper. The outbreak of the COVID-19 pandemic triggered the nationwide school closure, and hence the total disruption of school feeding services, ultimately expected to adversely affect households’ food security.

### Variable measurements: key explanatory variables

#### Access to (disruption of) school feeding services

As noted above, Nigeria closed all schools and introduced social distancing and mobility restrictions in mid-March 2020 (28). School closures disrupted school feeding services, limiting students’ access to school-based meal programs that contributed directly to households’ food security. We defined access to school feeding services using an indicator variable for those LGAs that ran school feeding services before the pandemic: those LGAs running school feeding services before the outbreak of the pandemic assumed a value of 1 and those LGAs not providing these services assumed a value of 0. Communities with school feeding services are those affected by COVID-19-induced disruptions to school feeding services, while communities without school feeding services are not. Thus, the indicator variable for access to school feeding services is equivalent to an indicator variable we generated for disruption of school feeding services. In January 2020, 314 of 368 LGAs in our sample were running school feeding services.

#### Primary school children

The NHGSFP provides 1 meal per day for each primary school child (grades 1–3) in a government-owned primary school in the implementing LGA. Thus, we also generated indicator variables for those households with primary school children, those households with children above primary school age (grades 4 and higher), and those without children. We defined primary school children using the age of the child and information on school participation; hence, those households with 1 or more school-going children aged 6–9 y are potential beneficiaries of school feeding services. We expected that disruption of school feeding services will only affect those households with primary school children. This targeting criterion of the school feeding program allowed us to conduct a falsification test to gauge the empirical relevance of possible violations of the identifying assumption. Under normal circumstances, those households without children and those with children older than primary school are not expected to be affected by disruption of school feeding services, although they might be affected by other effects of the pandemic.

#### COVID-19 cases and government lockdown measures

We measured the COVID-19-related infection incidence using the number of confirmed COVID-19 cases per million population in each state, considering all 37 states in Nigeria. [Nigeria has 36 states and 1 federal territory (the FCT of Abuja). For simplicity, we refer to these as 37 states.] We defined a dummy variable that assumes a value of 1 for those states within the last tercile of COVID-19 cases (that is, 65.2 COVID-19 cases per million population and above) and 0 for states in the first 2 terciles (those reporting below 65.2 COVID-19 cases per million population). As our post-COVID-19 survey was fielded in April and May 2020, we extracted confirmed COVID-19 cases until the end of May 2020. We are aware that the number of confirmed COVID-19 cases may underestimate the true infection rates in developing countries because of limited testing capacity. However, we believe that households and governments are likely to respond to these confirmed numbers, implying that these officially reported cases provide important information on the spread of the pandemic, as well as on the associated household and government responses.

As part of the measures to contain the spread of the pandemic, the federal- and state-level governments introduced various measures, including travel bans, closure of schools and religious institutions, bans on public and social gatherings, and curfew hours restricting the movement of people. To understand the implication of these government restrictions, we considered the strictest measures and mobility restrictions: that is, lockdown measures introduced by the federal and state governments of Nigeria (28, 31–33). We generated an indicator variable that takes a value of 1 for those states introducing lockdown measures to contain the spread of the virus and 0 otherwise. (The timing and length of lockdowns vary across states.) As our postpandemic survey was fielded in April and May 2020 and our food security questions elicited information on food insecurity experiences in the last 30 days, we considered lockdown measures introduced up until 15 May 2020.

To better understand the differential impacts of disruption of school feeding services on households' food security, we examined interactions between access to school feeding services and households’ baseline characteristics. We expected that poorer households, those with single mothers, and those living in remote areas were likely to be disproportionally affected by the pandemic and associated disruptions of school feeding services. We defined an indicator variable for poor households that assumed a value of 1 for households in the lowest asset tercile and 0 otherwise. We measured remoteness using distance to the state capital; those households living in areas farther than the median distance were defined as being “remote.”

### Variable measurements: outcome variables

#### Food insecurity indicators

We employed 3 indicators of food insecurity experience and a fourth, aggregate measure constructed using these indicators. Both survey rounds elicited information about households’ experiences of food insecurity and food shortages in the last 30 days. These indicators are commonly used to measure food security in other surveys and studies (34–36). The first indicator elicits whether an adult member of the household had to skip a meal because there was not enough money or other resources to get food. The second indicator elicits whether the household had run out of food in the last 30 days, mainly because there was not enough money or other resources to get food. Similarly, the third indicator assumes a value of 1 for those households whose adult member(s) went without eating for a whole day because of a lack of money or other resources, and assumes a value of 0 otherwise. These 3 measures are interlinked. Thus, we used a principal component analysis to construct an aggregate index as a fourth measure. We then standardized this index to facilitate interpretation. This food insecurity index is a linear combination of the 3 indicators of food insecurity experience. All our estimations employ these 4 indicators of food insecurity. Note that our food security indicators do not focus on children's food and nutrition security, but rather on household members’ food security. Although the food security situations of individual household members, including children, are likely to be strongly correlated, our outcome measures are not ideal for measuring and identifying the intrahousehold impacts of the pandemic and associated disruptions of school feeding services. Indeed, a growing body of literature argues that in the context of imminent food insecurity, adult members of households are likely to buffer younger household members against the adverse effects of food insecurity (37, 38).

## Statistical analysis

We aimed to quantify the adverse implications of disruption of school feeding programs on households’ food security by comparing the food security outcomes of beneficiary and nonbeneficiary households before and after the pandemic. We used the following difference-in-difference specification:

(1) \begin{eqnarray*} {Y_{hvt}} &=& {\alpha _h} + {\gamma _0}Pos{t_t}\nonumber\\ && +\, {\gamma _1}\ Disruption\ of\ school\ feedin{g_v}*Pos{t_t} + {\epsilon _{hvt}} \end{eqnarray*}

Here, ${Y_{hvt}}$ stands for the food insecurity outcome for each household (*h*) living in an LGA (*v*) and surveyed in round *t*. ${\alpha _h}$ stands for household fixed effects, which can capture all time-invariant heterogeneities across households. $Pos{t_t}$ is an indicator variable that assumes a value of 1 for the post-COVID-19 round and 0 for the pre-COVID-19 round. This time dummy captures aggregate trends in food security, as well as potential differences in our outcomes of interest driven by differences in survey methods (face-to-face or phone survey). $Disruption\ of\ school\ feedin{g_v}$ is an indicator variable assuming a value of 1 for those LGAs implementing school feeding services before the pandemic and 0 for those LGAs without school feeding services. Note that because of the total disruption of school feeding services triggered by the nationwide school closure, those communities with school feeding services are affected by COVID-19-induced disruptions of school feeding services, while those communities without school feeding services are not. ${\epsilon _{hvt}}\ $is an error term that absorbs any remaining unobservable factors that may generate variations in food insecurity. The interaction term and associated coefficient ${\gamma _1}\ $captures potential differential trends in food insecurity outcomes between those households that used to receive school feeding services and those not benefiting from the program. Thus, the specification in Equation *1* implements a standard difference-in-difference approach. As school feeding programs in Nigeria target primary school children (grades 1–3), we estimated the expression in Equation *1* for different households: those with primary school-going children (grades 1–3), those without children, and those with children above primary school age (grades 4 and above). Estimations for households without children or with children above primary school age are used as falsification tests to validate our causal inference on the effects of disruptions of school feeding services, as these households were not expected to be affected.

The impacts of school closure, and hence disruption of school feeding services, are likely to increase with the spread of the pandemic and associated lockdown measures. This is likely, as the spread of the pandemic (as measured by confirmed COVID-19 cases) and associated mobility restrictions may lead to a reduction in income-generating activities. To quantify the implications of the spread of the pandemic (intensity of COVID-19 cases), we examined the interactions between disruption of school feeding services and intensity of cases using an indicator variable for those states with a high number of COVID-19 cases. We expected that those states experiencing a higher rate of cases during the pandemic were more likely to witness a higher increase in food insecurity. We also quantified the implications of lockdowns by examining interactions between lockdown measures and disruption of school feeding services.

Note that we do not have information on household-level access to school feeding services, but rather information on LGA-level access. Thus, our estimates should be interpreted as intention to treat (ITT) measures (the impact of disruption of school feeding services in a community on those households living in a community) rather than actual treatment effects on the treated (ATT; the impact on actual beneficiaries of school feeding services). However, in the context of Nigeria, where most households send their children to government schools, the ITT estimates are not expected to be substantially different from the actual ATT estimates. In the worst case, our ITT estimates should be interpreted as lower bounds of the actual impact of disruptions of school feeding services on beneficiaries.

The impacts of the pandemic are likely to differ across households with varying socioeconomic statuses, including those with underlying vulnerabilities. For instance, poorer households are more likely to bear the brunt of disruptions of school feeding programs, as the poor are more likely to rely on school feeding services. Similarly, those households living in remote areas and those experiencing further disruptions of value chains and markets are expected to witness further deterioration in food security because of disruptions of school feeding services (39–41).

To account for potential systematic nonresponses in the post-COVID-19 phone survey, we weighted all our estimations using the sampling weight discussed. Using these sampling weights, we can recover appropriate and representative statistics, assuming that systematic nonresponses can be explained and captured using the long list of observable factors accounted for in the construction of weights (42, 43). Households living in the same LGA are likely to experience similar observable and unobservable services and shocks. Thus, we clustered standard errors at the LGA level, at which point access to school feeding services varied.

## Results

**Table 1** presents weighted summary statistics of selected household characteristics and key explanatory variables of interest, including access to school feeding services, confirmed COVID-19 cases, and government lockdown measures. As expected, no major differences arose in households’ observable characteristics across rounds. About 8% of our sample comprised single mothers in the pre- and postpandemic surveys. We defined a single mother as a mother who had a dependent child or dependent children and who was widowed, divorced, or unmarried. About three-fourths of the sampled households had school-going children. Almost half (47%) of households had at least 1 child of primary school age (6–9 y) and school-going children. About 83% of the sampled households lived in LGAs with school feeding programs. The mean number of state-level COVID-19 cases (at the end of May 2020) was 25.59 cases per million population, and about one-half of the states imposed lockdown restrictions in the period of 28 March through 15 May 2020.

**TABLE 1:** Selected characteristics of the study population in the pre-COVID-19 and post-COVID-19 rounds

	Pre-COVID-19 (2019)	Post-COVID-19 (2020)
Age of head, y	49.64 (14.73)	49.42 (14.24)
Education of head, y	8.21 (5.88)	8.87 (5.93)
Single mother, yes = 1	0.08 (0.20)	0.08 (0.20)
Family size, number	5.53 (3.81)	5.52 (3.32)
Value of assets, PPP US	1677.66 (3332.77)	N/A
Households with school-going children	0.74 (0.43)	N/A
Households with primary school age (6–9 y) school-going children	0.47 (0.49)	N/A
Household lives in local government area with school feeding program	0.83 (0.38)	N/A
Mean state-level COVID-19 cases, per million population	25.59 (97.74)	N/A
Lockdown measure, yes = 1	0.46 (0.49)	N/A
Poor households, poorest asset tercile = 1	0.33 (0.46)	N/A
Distance to state capital, km	56.05 (43.13)	N/A
*n*	1906	1906

Summary statistics are based on Nigeria LSMS-ISA 2019 and 2020 rounds of household data. Sample weights were applied. All values outside parentheses are means. SDs are given in parentheses. Abbreviations: COVID-19, coronavirus disease 2019; LSMS-ISA, Living Standards Measurement Study-Integrated Agriculture Survey; N/A, not available; PPP, purchasing power parity.

**Table 2** reports summary statistics of key outcome indicators on households’ food insecurity experiences in the last 30 d. The results show a significant increase in all food insecurity indicators. For example, the incidences of skipping a meal, running out of food, and going without eating increased by 47%, 32%, and 20%, respectively. This could be attributed to the spread of the pandemic and associated government restrictions that disrupted livelihood activities. For instance, Frongillo et al. (37) show that about 72% of households reported reduced incomes from farming and agricultural activities, 83% of households reported reduced income from nonfarm businesses, and about 50% of households reported reduced wage-related incomes.

**TABLE 2:** Means and mean differences in food security indicators between the pre-COVID-19 and post-COVID-19 rounds

Key outcome variables	Pre-COVID-19 (2019)	Post-COVID-19 (2020)	Mean difference (2020–2019)
Food security indicators^1^			
Skip a meal, 0/1	0.26	0.73	0.47^2^ (0.44–0.50)
Run out of food, 0/1	0.25	0.57	0.32^2^ (0.29–0.35)
Went without eating for a whole day, 0/1	0.05	0.24	0.20^2^ (0.18–0.22)
Food insecurity index, standardized PCA index^3^	−0.42	0.43	0.85^2^ (0.70–0.90)
*n*	1906	1906	—

Summary statistics are based on Nigeria LSMS-ISA 2019 and 2020 rounds of household data. The values in the first and second columns are means, while the values in the third column stand for mean differences. 95% CI values are given in parentheses. Abbreviations: COVID-19, coronavirus disease 2019; LSMS-ISA, Living Standards Measurement Study-Integrated Agriculture Survey; PCA, principal component analysis.

1 Food security indicators are measured as household-level responses to a question that elicits food insecurity experienced in the last 30 d.

2 *P* < 0.01.

3 The food insecurity index is constructed using PCA and standardized to have a (aggregate) mean of 0 and standard deviation of 1.

**Table 3** reports a descriptive comparison of trends in food insecurity indicators across different groups of households. Table 3 shows that households living in LGAs with school feeding services were more likely to experience a larger deterioration in food security in the latest (post-COVID-19) survey round. For instance, households living in LGAs with a school feeding program and with primary school children reported 8 and 7 percentage point higher changes in their experiences of skipping a meal and running out of food, respectively, in the last 30 d, when compared with households living in LGAs with no school feeding services. Most importantly, the changes in food security across rounds were larger for those households living in LGAs with school feeding services and those with primary school children. For those households with no school children or those with children above primary school, we did not observe significant differences across households with and without access to school feeding services. Our statistical analysis formally tested whether these differences were statistically significant and whether these increases in food insecurity could be attributed to school closures and disruption of school feeding services associated with the outbreak of the pandemic.

**TABLE 3:** Means and mean differences in households’ food security indicators across LGAs with and without school feeding programs for various types of households (those with and without primary school children)

	Households in LGAs with school feeding service			Households in LGAs with no school feeding service		
	Pre-COVID	Post-COVID	Mean difference	Pre-COVID	Post-COVID	Mean difference
Households with primary school children						
Skip a meal	0.21	0.72	0.51^1^ (0.47–0.55)	0.30	0.73	0.43^1^ (0.32– 0.53)
Run out of food	0.22	0.55	0.33^1^ (0.30–0.39)	0.34	0.60	0.26^1^ (0.14–0.36)
Went without eating for a whole day	0.04	0.27	0.23^1^ (0.19–0.26)	0.07	0.28	0.21^1^ (0.12–0.29)
Food insecurity index	−0.50	0.42	0.92^1^ (0.83–1.01)	−0.25	0.48	0.73^1^ (0.52–0.95)
*n*	746	746	—	151	151	—
Households with children above primary school age only						
Skip a meal	0.28	0.71	0.43^1^ (0.36–0.51)	0.32	0.76	0.44^1^ (0.29–0.60)
Run out of food	0.25	0.54	0.29^1^ (0.22–0.37)	0.35	0.62	0.27^1^ (0.08–0.42)
Went without eating for a whole day	0.06	0.25	0.19^1^ (0.13–0.24)	0.06	0.25	0.19^1^ (0.06–0.31)
Food insecurity index	−0.39	0.39	0.77^1^ (0.63–0.93)	−0.20	0.54	0.74^1^ (0.41–1.05)
*n*	305	305	—	65	65	—
Households with no school children						
Skip a meal	0.27	0.74	0.47^1^ (0.40–0.54)	0.27	0.73	0.46^1^ (0.33–0.60)
Run out of food	0.24	0.57	0.33^1^ (0.25–0.39)	0.25	0.59	0.34^1^ (0.20–0.48)
Went without eating for a whole day	0.04	0.18	0.14^1^ (0.09–0.18)	0.06	0.26	0.20^1^ (0.09–0.30)
Food insecurity index	−0.42	0.41	0.83^1^ (0.70–0.97)	−0.40	0.47	0.87^1^ (0.60–1.15
*n*	341	341	—	91	91	—

Summary statistics are based on Nigeria LSMS-ISA 2019 and 2020 rounds of household data. All values, except those in the third and sixth columns, are means. Those values in the third and sixth columns are mean differences (changes) across rounds. 95% CI values are given in parentheses. Abbreviations: LGA, local government area; LSMS-ISA, Living Standards Measurement Study-Integrated Agriculture Survey.

1 *P* < 0.01.

Our statistical analysis quantified the impact of disruption of school feeding services associated with school closures on households’ food security for households that were expected to be affected by these disruptions and those that were not. We also examined whether such impacts increased with the spread of the pandemic and government-imposed mobility restrictions, which were shown to adversely affect livelihood and income-generating activities in Nigeria (44). Finally, we documented potential heterogenous impacts of such disruptions of school feeding services across various groups of households.

### Impact of disruption of school feeding services on food security

**Table 4** shows the impacts of disruption of school feeding services for those households with primary school children, with children above primary school, and without school children, and 3 important findings stand out. First, for all types of households, reports of food insecurity experience increased substantially in the post-COVID-19 survey. This is consistent with the descriptive evidence reported in Table 2 and Table 3. Second, disruptions of school feeding services increased the food insecurity experiences of households with primary school children. The interaction between disruption of school feeding services and the postpandemic dummy captures the temporal variation in the evolution of our outcomes of interest across households with varying exposure to school feeding services. Third, as expected, disruption of school feeding services only impacted households with primary school children. For example, the results for those households with primary school children in Table 4 show that the disruption of school feeding services associated with the spread of the pandemic increased the probability that a household with primary school children skipped a meal in the last 30 d by 9 percentage points. Similarly, disruption of school feeding services was associated with a 0.2 SD increase in the food insecurity index. That is, households with primary school-going children were more likely to experience further deterioration in food security due to the disruption of school feeding services. Intuitively, such impacts are not statistically significant for households with children above primary school and for those households without school children, which suggest that the impacts are likely to be causal impacts of COVID-19-induced disruption of school feeding services on food security. The consistent patterns documented across alternative measures of food insecurity indicators strengthen our findings and causal claim.

**TABLE 4:** Difference-in-differences estimations of the impact of disruption of school feeding services on household food security, by households’ exposure to school feeding services (school status of their children)

	(9)	(10)	(11)	(4)
	Skipped a meal, coefficients (95% CI)	Ran out of food, coefficients (95% CI)	Went without eating for a whole day, coefficients (95% CI)	Food insecurity index, coefficients (95% CI)
Households with primary school children				
Postpandemic dummy, 2020 round	0.43^1^	0.25^1^	0.20^1^	0.73^1^
	(0.35–0.50)	(0.14–0.36)	(0.11–0.29)	(0.54–0.91)
Disruption of school feeding^2^ *Post	0.09^3^	0.09	0.03^4^	0.20^3^
	(0.03–0.17)	(−0.02 to 0.21)	(0.02–0.11)	(0.04–0.41)
Constant	0.23^1^	0.24^1^	0.04^1^	−0.46^1^
	(0.20–0.25)	(0.21–0.26)	(0.03–0.06)	(−0.50 to −0.42)
Household fixed effects	Yes	Yes	Yes	Yes
R-squared	0.42	0.23	0.18	0.37
*n*	1794	1794	1794	1794
Households with children above primary school age only				
Postpandemic dummy, 2020 round	0.45^1^	0.25^1^	0.18^1^	0.73^1^
	(0.32–0.57)	(0.11–0.38)	(0.07–0.30)	(0.49–0.98)
Disruption of school feeding*Post	−0.01	0.05	0.01	0.06
	(−0.15 to 0.13)	(−0.10 to 0.19)	(−0.12 to 0.13)	(−0.22 to 0.33)
Constant	0.28^1^	0.27^1^	0.06^1^	−0.36^1^
	(0.25–0.32)	(0.24–0.30)	(0.03–0.08)	(−0.42 to −0.30)
Household fixed effects	Yes	Yes	Yes	Yes
R-squared	0.34	0.18	0.13	0.29
*n*	740	740	740	740
Households with no school children				
Postpandemic dummy, 2020 round	0.46^1^	0.35^1^	0.21^1^	0.88^1^
	(0.30–0.61)	(0.23–0.46)	(0.11–0.31)	(0.64–1.12)
Disruption of school feeding*Post	0.02	−0.02	−0.07	−0.04
	(−0.15 to 0.19)	(−0.16 to 0.11)	(−0.18 to 0.04)	(−0.32 to 0.23)
Constant	0.27^1^	0.24^1^	0.04^1^	−0.42^1^
	(0.23–0.30)	(0.22–0.27)	(0.02–0.07)	(−0.48 to −0.36)
Household fixed effects	Yes	Yes	Yes	Yes
R-squared	0.39	0.23	0.10	0.35
*n*	864	864	864	864

Estimations are based on Nigeria LSMS-ISA 2019 and 2020 rounds of household data. All estimations are adjusted by sampling weights to account for nonresponses in the phone survey. Values out of parenthesis are associated with the coefficients described in Equation *1*. Values in parentheses are 95% CIs. Using households’ school children status, we classify households into 3 groups: households with primary school children, with children above primary school age, and with no school children. Abbreviations: LGA, local government area; LSMS-ISA, Living Standards Measurement Study-Integrated Agriculture Survey.

^1^ *P* < 0.01.

2 Disruption of school feeding is an indicator variable assuming a value of 1 for those households living in LGAs implementing school feeding services before the pandemic and 0 for those households in LGAs without school feeding services.

^3^ *P* < 0.05.

4 *P* < 0.10.

### Role of the spread of the pandemic and government responses

The results in the first few rows of **Table 5** show that the disruption of school feeding services had a higher impact for households residing in states experiencing higher COVID-19 cases. This is in addition to the overall and aggregate effects of the spread of the pandemic among all households. This likely reflects that in addition to the disruption of school feeding services, the spread of the pandemic limited income-generating activities, which further led to deterioration of food security. The remaining results in Table 5 (those related to the implications of lockdown measures) show that disruption of school feeding programs had a higher impact for households in states that introduced the strictest mobility restriction (lockdown) measures. Indeed, these results show that disruption of school feeding services had a negligible impact in those states with no lockdown measures. For example, the first column estimates show that those households experiencing both disruption of school feeding services and strict lockdown measures reported an additional increase in their experience of food insecurity (probability of skipping a meal). Similarly, these results show that disruption of school feeding services along with lockdown measures was associated with an additional increase in the food insecurity index.

**TABLE 5:** Difference-in-differences estimations of the impact of the intensity of COVID-19 and lockdown measures on households’ food security (for those households with primary school children)

	(9)	(10)	(11)	(4)
	Skipped a meal, coefficients (95% CI)	Ran out of food, coefficients (95% CI)	Went without eating for a whole day, coefficients (95% CI)	Food insecurity index, coefficients (95% CI)
COVID-19 cases and school feeding services				
Postpandemic dummy, 2020 round	0.41^1^	0.23^1^	0.20^1^	0.69^1^
	(0.33–0.48)	(0.13–0.34)	(0.11–0.28)	(0.50–0.88)
Disruption of school feeding*Post	0.07	0.08	0.02	0.16
	(−0.02 to 0.16)	(−0.04 to 0.20)	(−0.08 to 0.12)	(−0.05 to 0.37)
Disruption of school feeding*Post*High cases^2^	0.08^3^	0.07	0.03^3^	0.16^3^
	(0.02–0.16)	(−0.02 to 0.16)	(0.00–0.11)	(0.00–0.35)
Constant	0.23^1^	0.24^1^	0.04^1^	−0.46^1^
	(0.20–0.25)	(0.21–0.26)	(0.03–0.06)	(−0.50 to −0.42)
Household fixed effects	Yes	Yes	Yes	Yes
R-squared	0.42	0.23	0.18	0.37
*n*	1794	1794	1794	1794
Lockdown measures and school feeding services				
Postpandemic dummy, 2020 round	0.43^1^	0.25^1^	0.20^1^	0.73^1^
	(0.35–0.50)	(0.14–0.36)	(0.11–0.29)	(0.54–0.91)
Disruption of school feeding*Post	−0.01	0.03	0.04	0.04
	(−0.12 to 0.10)	(−0.11 to 0.16)	(−0.08 to 0.15)	(−0.20 to 0.29)
Disruption of school feeding*Post*Lockdown^4^	0.15^1^	0.11^5^	−0.03	0.25^5^
	(0.05–0.25)	(0.01–0.21)	(−0.11 to 0.06)	(0.04–0.45)
Constant	0.23^1^	0.24^1^	0.04^1^	−0.46^1^
	(0.21–0.25)	(0.21–0.26)	(0.03–0.06)	(−0.50 to −0.42)
Household fixed effects	Yes	Yes	Yes	Yes
R-squared	0.42	0.23	0.18	0.37
*n*	1794	1794	1794	1794

Estimations are based on Nigeria LSMS-ISA 2019 and 2020 rounds of household data. All estimations are adjusted by sampling weights to account for nonresponses in the phone survey. Values out of parentheses are associated with the coefficients described in Equation *1*. Values in parentheses are 95% CIs. Abbreviations: COVID-19, coronavirus disease 2019; LSMS-ISA, Living Standards Measurement Study-Integrated Agriculture Survey.

1 *P* < 0.01.

2 We defined a dummy variable for states with high COVID-19 cases (an indicator variable assuming a value of 1 for those states within the last tercile of COVID-19 cases and 0 for other states).

3 *P* < 0.10.

4 Lockdown is an indicator variable for those states that introduced lockdown measures to contain the spread of the virus.

^5^ *P* < 0.05.

#### Heterogenous impacts of disruption to school feeding programs

The results in **Table 6** show the heterogeneous impact of the disruption of school feeding services on households’ food security. The first panel estimates show that single mothers with primary school-going children were likely to report a higher increase in the probability of skipping a meal or running out of food in the last 30 d. Similarly, poorer households with primary school-going children were likely to report an 11–percentage point higher probability of skipping a meal in the last 30 d. Poorer households are more likely to rely on school feeding services for accessing nutritious diets and are likely to be disproportionally affected by the closure of school feeding programs.

**TABLE 6:** Difference-in-differences estimations of heterogenous impacts of disruption of school feeding services on households’ food security, by households’ socioeconomic status (for those households with primary school children)

	(9)	(10)	(11)	(4)
	Skipped a meal, coefficients (95% CI)	Ran out of food, coefficients (95% CI)	Went without eating for a whole day, coefficients (95% CI)	Food insecurity index, coefficients (95% CI)
Postpandemic dummy, 2020 round	0.43^1^	0.25^1^	0.21^1^	0.73^1^
	(0.36–0.51)	(0.14–0.35)	(0.12–0.30)	(0.55–0.91)
Disruption of school feeding*Post*single mother^2^	0.08^3^	0.10^3^	0.02	0.20^4^
	(0.01–0.17)	(−0.01 to 0.22)	(−0.08 to 0.12)	(0.03–0.41)
Constant	0.23^1^	0.24^1^	0.04^1^	−0.46^1^
	(0.20–0.25)	(0.21–0.26)	(0.03–0.06)	(−0.50 to −0.42)
Household fixed effects	Yes	Yes	Yes	Yes
*n*	1794	1794	1794	1794
Postpandemic dummy, 2020 round	0.47^1^	0.32^1^	0.20^1^	0.86^1^
	(0.43–0.52)	(0.28–0.37)	(0.16–0.24)	(0.77–0.96)
Disruption of school feeding*Post*asset-poor tercile^5^	0.11^4^	0.02	0.11^4^	0.15
	(0.01–0.20)	(−0.09 to 0.13)	(0.02–0.19)	(−0.06 to 0.36)
Constant	0.23^1^	0.24^1^	0.04^1^	−0.46^1^
	(0.21–0.25)	(0.21–0.26)	(0.03–0.06)	(−0.50 to −0.42)
Household fixed effects	Yes	Yes	Yes	Yes
*n*	1794	1794	1794	1794
Post-pandemic dummy, 2020 round	0.46^1^	0.29^1^	0.20^1^	0.82^1^
	(0.41–0.52)	(0.23–0.36)	(0.15–0.25)	(0.69–0.94)
Disruption of school feeding*Post*remote^6^	0.07^3^	0.07^3^	0.04	0.17^4^
	(−0.01 to 0.15)	(−0.01 to 0.16)	(−0.03 to 0.11)	(0.00–0.33)
Constant	0.23^1^	0.24^1^	0.04^1^	−0.46^1^
	(0.20–0.25)	(0.21–0.26)	(0.03–0.06)	(−0.50 to −0.42)
Household fixed effects	Yes	Yes	Yes	Yes
*n*	1794	1794	1794	1794

Estimations are based on Nigeria LSMS-ISA 2019 and 2020 rounds of household data. All estimations are adjusted by sampling weights to account for nonresponses in the phone survey. Values outside parentheses are associated with the coefficients described in Equation *1*. Values in parentheses are 95% CIs. Abbreviation: LSMS-ISA, Living Standards Measurement Study-Integrated Agriculture Survey.

1 *P* < 0.01.

2 We defined a single mother as a mother who has a dependent child or dependent children and who is widowed, divorced, or unmarried.

3 *P* < 0.10.

4 *P* < 0.05.

5 Poor households are those with a value of assets falling in the first tercile.

6 Remote households are those living in remote areas: that is, those living in areas farther than the median distance to the state capital.

## Discussion

This study combined nationally representative, pre-COVID-19, in-person survey data with postpandemic phone survey data, along with disaggregate information on access to school feeding services, to quantify the impacts of COVID-19 and associated disruptions of school feeding services on households’ food security in Nigeria. We aimed to shed light on and identify the differential impacts of the pandemic and associated disruptions of school feeding services. Consistent with recent studies, we found that food security deteriorated for most households, but more so for those that previously benefited from school feeding services. Most importantly, we found that the COVID-19-induced disruption of school feeding services had significant impacts on the food security of beneficiary households. Disruption of school feeding services increased households’ experiences of food insecurity (probability of skipping a meal) by 9 percentage points (*P <* 0.05). This suggests that the pandemic had a significant adverse impact among Nigerian households, as also documented by the significant increase in income loss, both in Nigeria (44) and in other African countries (45, 46). We showed that these impacts are only statistically significant for those households with primary school children (grades 1–3). Falsification tests based on those households without school-going children and with children above primary school age supported our causal claim on the impact of disruptions of school feeding programs on beneficiary households. These impacts increased with the spread of the pandemic and the government's associated mobility restrictions, suggesting that the spread of COVID-19 and lockdown measures further exacerbated food insecurity by limiting livelihood and income-generating activities.

We also found that single mothers, poorer households, and those living in remote areas experienced relatively larger deteriorations in food security because of the disruptions of school feeding services. This is intuitive because working and single mothers are more likely to bear the brunt of increased childcare needs associated with school and daycare center closures (20, 21). Similarly, poorer households are more likely to rely on school feeding services for acquiring nutritious food for their children and may thus experience further deterioration in food security. Previous studies have shown that the impact of school feeding services on improving food security, and hence reducing poverty, is larger for poorer households (18).

These findings have important implications in terms of both identifying additional impacts of the COVID-19 pandemic and highlighting the food security impacts of school feeding services. In addition to the direct health and income impacts identified, we found that disruption of education and nutritional services has endangered households’ food security in Nigeria. The COVID-19 pandemic is an ideal natural experiment to quantify the role of school feeding services, and our findings provide additional evidence on the potential of school feeding programs to improve households’ food security (10, 13, 16, 17, 35). The heterogeneous impacts of the disruption of school feeding services corroborate evolving studies arguing that the pandemic has increased existing gender inequalities (1, 20, 21) and income inequalities. These findings can help inform immediate and medium-term policy responses, including the design of alternative social protection policies and nutritional services to mitigate longer-term adverse economic and welfare implications. Furthermore, our findings can inform future investment options and nutrition-sensitive interventions to facilitate and ensure sustainable recovery. The heterogenous impacts documented in this study can help governments and international donor agencies improve their targeting strategies to identify the most impacted subpopulations and households.

Despite our attempt to shed light on an important impact of the COVID-19 pandemic, this study suffered from some limitations. First, phone surveys are not well suited for collecting detailed information on household members’ consumption, so we were not able to identify intrahousehold impacts and differences. Future studies will hopefully improve these data limitations and provide additional evidence of the intrahousehold effects of the COVID-19 pandemic. Second, we did not have the privilege and benefits of randomized variations in access to school feeding services. Cognizant of this, we controlled for household fixed effects and conducted several falsification tests to demonstrate that our results were driven by other confounding factors. These fixed effects capture all time-invariant differences between communities with and without school feeding services, while other unobservable differential trends between these communities may still bias our results. Third, our food security measures were based on a few critical questions capturing the most severe food insecurity, rather than the full set of questions forming the Food Insecurity Experience Scale (FIES). Given the context and the severest level of food insecurity in Nigeria, these questions were expected to reasonably capture households’ food security status, but a full set of FIES questions would likely provide additional insights on various domains of food insecurity. Finally, we lacked actual information on households’ access to school feeding services, and hence relied on subdistrict-level information to define our treatment variable. Future studies may revisit this using detailed household-level information on households’ access to school feeding services.

## Notes

Author disclosures: This paper has been prepared as an output of the Nigeria Strategy Support Program, which is managed by the International Food Policy Research Institute (IFPRI) and funded by the United States Agency for International Development (USAID) under the Feed the Future Nigeria Agricultural Policy Project. The research presented here was also funded by the CGIAR Research Program on Policies, Institutions, and Markets (PIM), which is led by IFPRI. KAA and LT acknowledge funding from the Partnership for Economic Policy (PEP), which is financed by the Department for International Development (DFID) of the United Kingdom (UK Aid) and the International Development Research Centre (IDRC) of Canada. MA and KSA, no conflicts of interest.

Abbreviations used: ATT, treatment effects on the treated; COVID-19, coronavirus disease 2019; FCT, Federal Capital Territory; FIES, Food Insecurity Experience Scale; ITT, intention to treat on the treated; LGA, local government area; NHGSFP, The National Home-Grown School Feeding Program; NSIP, National Social Investment Program.
